# Cost-effectiveness analysis of rotavirus vaccination in China: Projected possibility of scale-up from the current domestic option

**DOI:** 10.1186/s12879-016-2013-1

**Published:** 2016-11-15

**Authors:** Shuhui Cui, Ruoyan Gai Tobe, Xiuting Mo, Xiaoyan Liu, Lingzhong Xu, Shixue Li

**Affiliations:** 1 School of Public Health, Shandong University, Jinan, China; 2 Department of Health Policy, National Center for Child Health and Development, Okura 2-10-1, Setagaya-ku, Tokyo, 157-8535 Japan

**Keywords:** Rotavirus, Vaccination, China, Cost-effectiveness, Routine immunization

## Abstract

**Background:**

Rotavirus infection causes considerable disease burden of acute gastroenteritis (AGE) hospitalization and death among children less than 5 years in China. Although two rotavirus vaccines (Rotarix and RotaTeq) have been licensed in more than 100 countries in the world, the Lanzhou Lamb rotavirus vaccine (LLR) is the only vaccine licensed in China. This study aims to forecast the potential impacts of the two international vaccines compared to domestic LLR.

**Methods:**

An economic evaluation was performed using a Markov simulation model. We compared costs at the societal aspect and health impacts with and without a vaccination program by LLR, Rotarix or RotaTeq. Parameters including demographic, epidemiological data, costs and efficacy of vaccines were obtained from literature review. The model incorporated the impact of vaccination on reduction of incidence of rotavirus infection and severity of AGE indicated by hospitalization, inpatient visits and deaths. Outcomes are presented in terms of quality-adjusted life years (QALYs) gained and incremental cost-effectiveness ratio (ICER) compared to status quo.

**Results:**

In a hypothetical cohort of 100,000 infants, the two international vaccines showed very good cost-effectiveness, with ICER of Rotateq and Rotarix shifting from LLR of $1715.11/QALY and $2105.66/QALY, respectively. Rotateq and Rotarix had significantly decreased incidence compared to LLR, particularly among infants aged 6 months to 2 years.

**Conclusions:**

RotaTeq is expected to introduce in the national routine immunization program to reduce disease burden of rotavirus infection with universal coverage.

## Background

Diarrhea is the second leading cause of child mortality only after pneumonia in the world. Rotavirus is the most common cause of acute gastroenteritis (AGE) among children under 5 years of age and leads to substantial mortality and morbidities in both developing and developed countries [1]. Rotavirus causes approximately 111 million episodes of gastroenteritis requiring home care, leads to 25 million outpatients and 2 million hospitalizations [2]. Worldwide in 2008, diarrhea attributable to rotavirus infection resulted in 453,000 under-five deaths, accounting for 37 % of deaths attributable to diarrhea and 5 % of all deaths in children younger than 5 years [3]. In China, approximately 47.8 % of AGE hospitalization among under-five children attributed to rotavirus [4]. Although the overall annual number of deaths from rotavirus diarrhea decreased by 74 % during the past years, rural children suffer from rotavirus-related deaths 11 times greater than urban children (0.33 deaths vs. 0.03 deaths per live birth in 2012) [5]. There are annually 134,000 deaths of children under 5 years due to rotavirus infection and about 70 % of them living in the rural area [6]. Rotavirus diarrhea is currently no specific treatment. The main measures of oral rehydration salts (ORS) and intravenous fluids are to maintain electrolyte balance and to reduce the number of deaths caused by diarrheal dehydration. Therefore, vaccination against rotavirus serves as a principal strategy to reduce the disease burden.

There are two types in the world: Rotateq (a pentavalent vaccine manufactured by Merck, US) and Rotarix (a monovalent vaccine manufactured by GlaxoSmithKline, Belgium) and both have been approved in more than 100 countries, but not yet in China. Rather, a domestic Lanzhou Lamb Rotavirus vaccine (LLR) developed by Rotorway Lanzhou Institute of Biological Products has been licensed since 1998 and introduced into the second-category list of the national immunization program, which is not compulsory with free access and needs to charge a user-fee. The Rotarix vaccine is derived from a single human rotavirus strain (89–12; P[8] G1) that was attenuated by multiple passages in cell culture, divided into 2 doses of oral, interval 1 to 2 months [7]. The RotaTeq vaccine based on a parent bovine strain (WC3) is composed of 5 rotavirus strains that contains the types of rotavirus (G1–G4 and P1A) [7]. It need to take three doses of oral, each agent an interval of at least 1 month. The LLR vaccine is derived from a lamb rotavirus strain (P[12] G10),which induces generation the antigenic types of rotavirus (G1–G4), recommended to take one dose of oral annually [7]. The clinical research about 4000 infants and young children(aged 6 ~ 24 months), shows that the effectiveness of RV diarrhea protection was 78 % [8].

The World Health Organization (WHO) recommends introduction of the rotavirus vaccine into the routine immunization program, which can potentially bring health benefits including reduced hospitalization and mortality [9, 10]. Globally, rotavirus vaccination had been implemented in the national vaccination program of 75 countries at the moment. In China, a national routine immunization program is expected to reduce child mortality in rural areas and hospitalization in urban areas related to rotavirus. Although the national routine immunization program by RotaTeq and Rotarix have shown good cost-effectiveness in both developing and developed countries [11–13], there was seldom economic evaluation to forecast the potential impacts of the two international vaccines with comparison to the domestic vaccine in China. Therefore, the aim of this study is to assess cost-effectiveness of possible options of rotavirus vaccination in China, in order to inform the decision making.

## Methods

### Overview of the model

**Fig. 1:**
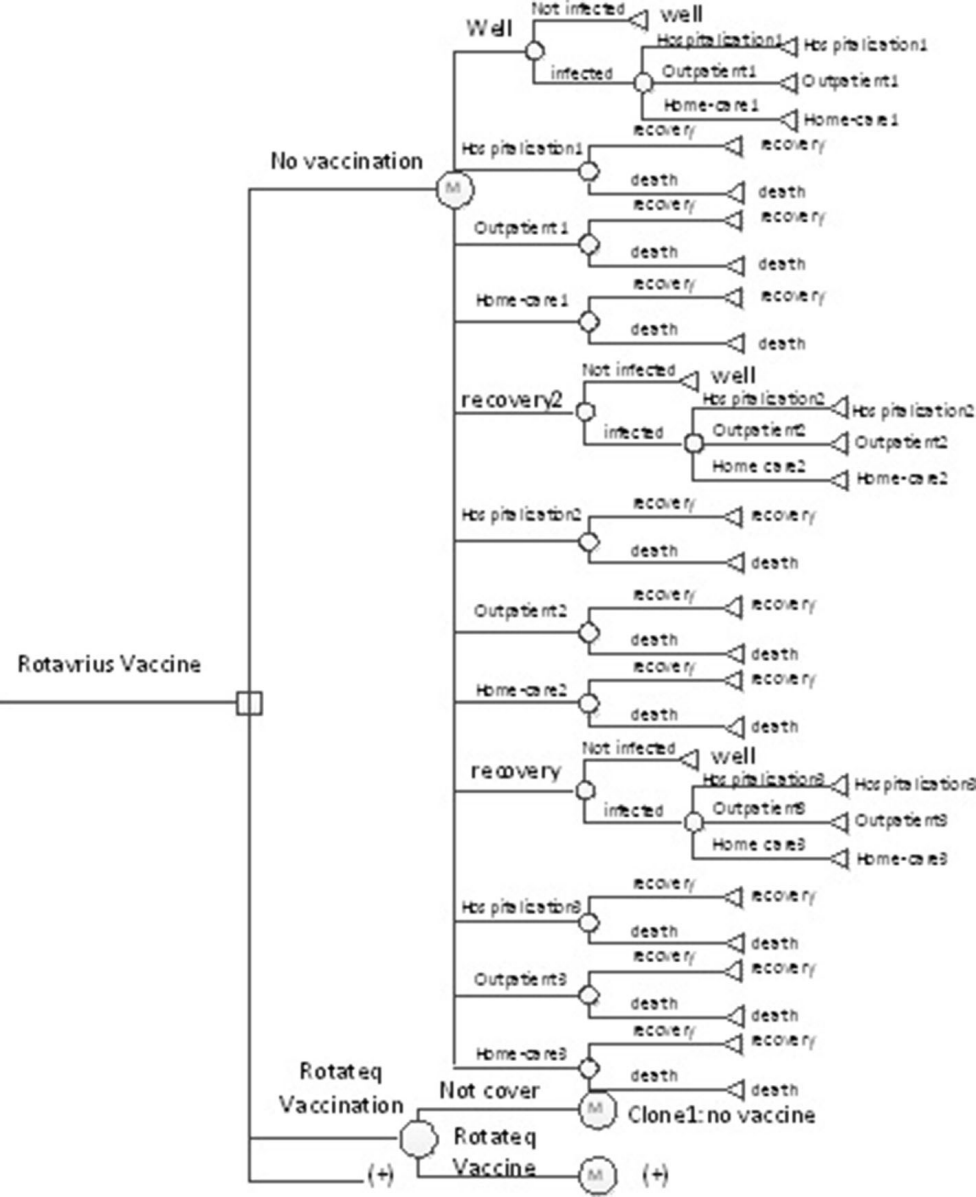
Decision tree-Markov model

We constructed a Markov model in TreeAge Pro 2011 (TreeAge Software Inc.) in a hypothetical birth cohort of 100,000 in the rural and the urban area to compare two international vaccines (Rotarix and Rotateq) with the current LLR and all vaccination options with no vaccination (Fig. 1). The model simulated 6 months for a cycle, following up until the age of 60 months, with the time horizon of the life span. At the initial point of each cycle, the birth cohort enters the model to either the vaccinated or the unvaccinated branch, and shift to different health status including staying healthy, acquiring rotavirus infection, or dying due to rotavirus-related diseases or other reasons. Children infected by rotavirus were considered to receive either inpatient or outpatient services, or stay at home, depending on the severity and the utilization of healthcare services. Infected individuals who did not die returned to a healthy status at the end of each cycle. The transition probabilities are specified by cycles, including immunization coverage, vaccine efficacy, and incidence, hospitalization and mortality of rotavirus-related diseases. The expected outcomes include reduction of the incidence and the severity of rotavirus infection and utilization of healthcare services.

### Cost estimates

Costs were given in 2015 prices in US dollars and discounted to the net present values using an annual rate of 3 %. Purchasing Power Parity (PPP) conversion rates and the domestic consumer price index (CPI) of health care were used to adjust costs data [14, 15] into US dollars. Cost estimates are based on societal perspective, including vaccination costs and medical costs to treat rotavirus-related diseases. We considered costs as the overall health resources consumed for vaccination or medical care, rather than payment by patients. Regarding vaccination costs, because the two international vaccines have not been approved in China yet, we referred the basic data across different countries worldwide and set a wide range of the price in sensitivity analysis [16, 17]. The total cost of a single dose of LLR was $24 based on the national tariff, as currently it is listed in the second-category vaccines and fully charged to users. Regarding the costs of rotavirus-associated inpatient and outpatient services, data were derived from a facility-based study of the economic burden associated with rotavirus diarrhea in five provinces of China [18] and a population-based surveillance for measuring the economic burden of the disease in a rural area of China [19]. We referred similar measurement of one of the previous economic evaluation by Liu et al to consider the different levels of healthcare in the country [20].

### Epidemiological data on the disease burden

In China, population-based estimated rates of rotavirus-related inpatient and outpatient was 14.4 and 149 per 100,000 under-five children, respectively [21]. The real incidence of the disease is likely to be underestimated because rotavirus was less commonly detected in children with mild cases in primary clinics and in the community [22]. By age five, it is assumed that almost every child will have an episode of rotavirus gastroenteritis, approximately 20 % will be outpatient, 1.67 % will be inpatient and 0.34 % will die [2]. We considered differences on the incidence and disease burden due to rotavirus infection in rural and the urban area of China.

First rotavirus infections are most likely to result in moderate-severe cases of rotavirus gastroenteritis but subsequent infections are progressively milder. The adjusted efficacy of a child’s first natural rotavirus infection in protecting against subsequent natural rotavirus-associated diarrhea was 77 %. This protection increased to 83 % after two natural infections and to 92 % after three natural infections [23].

The annual background mortality was based on data from the Hospital Statistics. Since there were very few RV-infections leading to death in China, the background mortality was not adjusted for RV-associated deaths. This data was adjusted to the aver-age population <5 years of age. Accordingly, the calculated the probability of each phase to die due to RV-infection was 0.0058 % [24].

### Vaccine effectiveness

The protection effectiveness of Rotarix and Rotateq were derived from randomized controlled trials in other Asian regions such as Hong Kong, Taiwan and Singapore, considering the ethinic homogeneity [25], because there was no eligible data specifically for the Chinese population. Both were highly effective in protecting against rotavirus gastroenteritis (RVGE) [25, 26]. Because there was no randomized control trial assessing LLR, we used data from a domestic meta-analysis to explore the protective effect [27] and two case-control studies to evaluate the vaccine effectiveness [28, 29].

### Outcome measures and cost effectiveness analysis

The health benefits of the vaccination include reduced rotavirus-related deaths, hospitalizations and outpatient visits and saved medical costs. The incremental cost-effectiveness ratio was calculated as incremental costs divided by incremental quality-adjusted lift year (QALY) gained, in order to determine the priority to purchasing the interventions at different budget levels. The health utility of different disease status were derived from previous literature [30]. A willingness-to-pay (WTP) thresholds of three times Gross Domestic Product (GDP) per capita was applied to examine the cost-effectiveness of the vaccination options, as WHO defined interventions with ICER less than three times of GDP per capita as cost-effective and that less than GDP per capita as very cost-effective [31].

### Sensitivity analysis

To test the robustness of model results, we varied the assumptions of parameters and a discount rate of 3 % for estimates of costs and health impact over a plausible range in sensitivity analysis (Table 1). Besides one-way sensitivity analysis, probabilistic multivariate sensitivity analysis with the Monte Carlo simulation of 100,000 randomly selected sets of net costs and health benefits were performed. For each set, the frequency distributions for every assumption were randomly sampled to assign assumption values.

**Table 1:** Parameters and plausible ranges in the model

	Baseline	Plausible range for sensitivity analysis		Sources
Parameters				
Discount rate	0.03	0	0.03	[31]
Vaccine coverage	25.3 %	10 %	28.6 %	[37, 36]
Mortality rate	0.0058 %	0.000029	0.000039	[41]
Rotateq efficacy	98 %	0	0.98	[38]
Rotateq infected	0.018 %	0	0.00018	[42]
hospitalization1^a^	44 %	0	0.44	[22]
Outpatient1^a^	28 %	0	0.28	[22]
Home-care1^a^	28 %	0	0.28	[22]
Rotarix infected	0.1 %	0	0.001	[26]
LLR infected	0.9 %	0	0.009	[41]
hospitalization3^c^	0.2 %	0	0.002	[2]
Outpatient3^c^	7.9 %	0	0.079	[2]
home-care3^c^	91.9 %	0	0.919	[2]
Rotarix efficacy	96.1 %	0.871	1	[25, 26]
LLR efficacy	72 %	0.63	0.79	[27]
Infection rate	78.85 %	0	0.7885	[34]
home-care2^b^	32 %	0	0.32	[22]
hospitalization2 ^b^	33 %	0	0.33	[22]
Outpatient2^b^	35 %	0	0.35	[22]
natural protact1^d^	77 %	0	0.77	[23]
natural protact2^d^	83 %	0	0.83	[23]
Costs				
International vaccinations	200.00	5	250	[16, 17]
LLR vaccination	24			The national tariff
Hospitalizations	570.04	0	570.04	[43]
Outpatient	104.19	0	104.19	[43]
Home-care	11.52	0	11.52	[44]
Health Effects				
Mortality rate	0.0058 %	0	0.000058	[24]
QALY(Hospitalization)	0.077	0.075	0.078	[30]
QALY(Outpatient)	0.081	0	0.081	[30]
QALY(Home-care)	0.082	0	0.082	[30]

^a^Children's first post-infection treatment method (Outpatient, hospitalization, and home-care) selection probabilities

^b^Children's second post-infection treatment method (Outpatient, hospitalization, and home-care) selection probabilities

^c^Children's third post-infection treatment method (Outpatient, hospitalization, and home-care) selection probabilities

^d^Children's own protective efficacy first cured after infection,and Children's own protective efficacy second cured after infection

## Results

### Health impacts and cost-effectiveness of vaccination

**Fig. 2:**
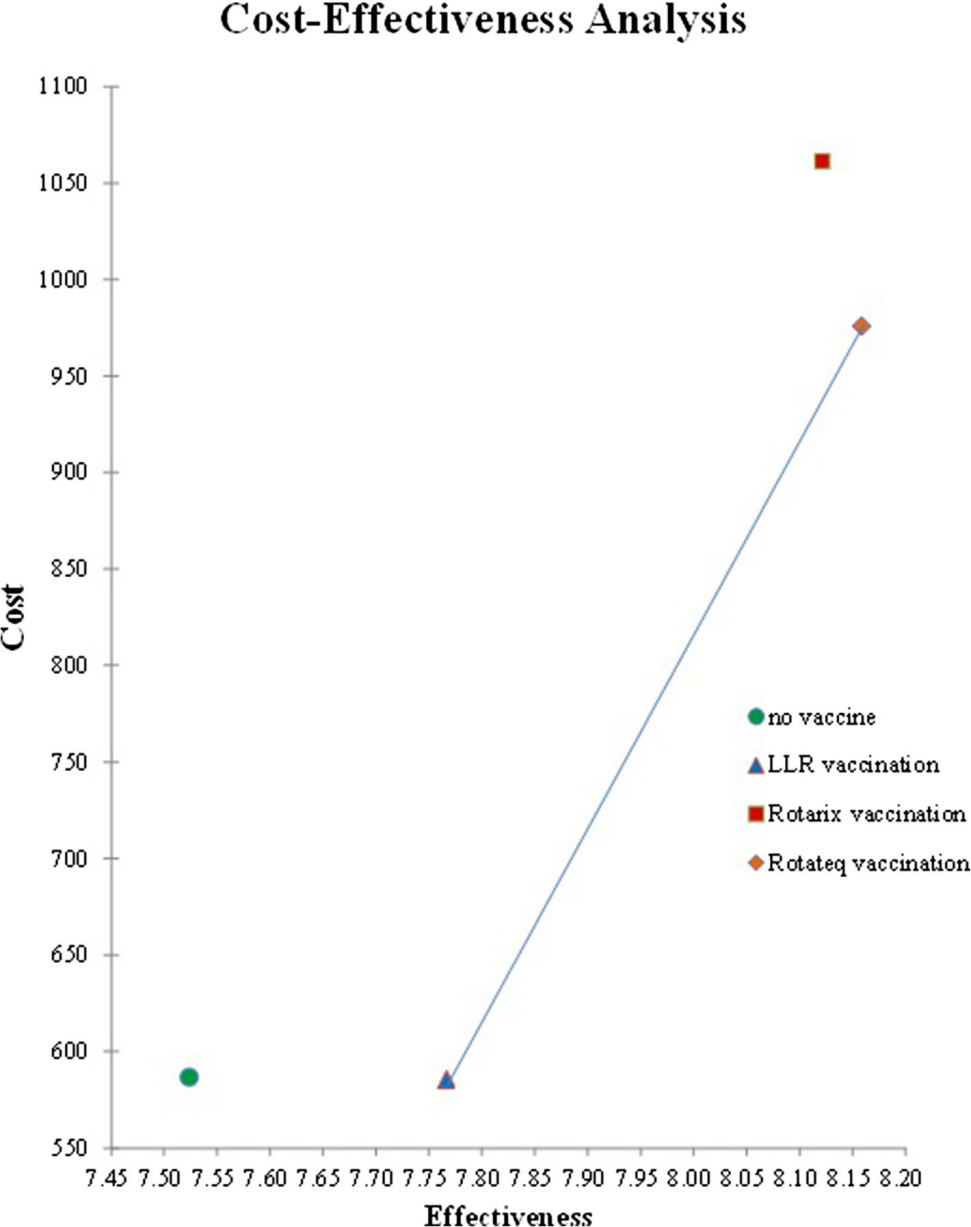
Cost-effectiveness of rotavirus vaccines at the baseline

In a hypothetical cohort of 100,000 infants, projected costs and health benefits for all vaccination options are shown in Table 2. Rotateq and Rotarix both showed very good cost-effectiveness, with ICER lower than GDP per capita. Of the three options, LLR is the basic one with average cost-effectiveness ratio (ACER) of $110.68/QALY gained. The total cost is even less than non-vaccination. Rotateq yielded the most health benefits, with ACER of $228.13/QALY gained (Fig. 2). The incremental cost-effectiveness ration (ICER) of Rotateq and Rotarix shifting from LLR was $1715.11/QALY gained and $2105.66/QALY gained, respectively, indicating that the optimal pathway for children rotavirus vaccination starts at LLR and then shift to Rotateq.

**Table 2:** Costs, health impacts and cost-effectiveness of rotavirus vaccines with comparison to no intervention

Strategy Name	Cost	QALYs	Incremental cost-effectiveness ratio ($/QALY)
No vaccine	7.524391	586.6089	−5.58
LLR vaccine	7.629827	586.0207	0
Rotarix vaccination	8.123035	1061.374	−2308.74
Rotateq vaccination	8.160051	975.9129	990.43

Ratio of additional costs and benefits of a particular strategy compared with the no intervention strategy

**Fig. 3:**
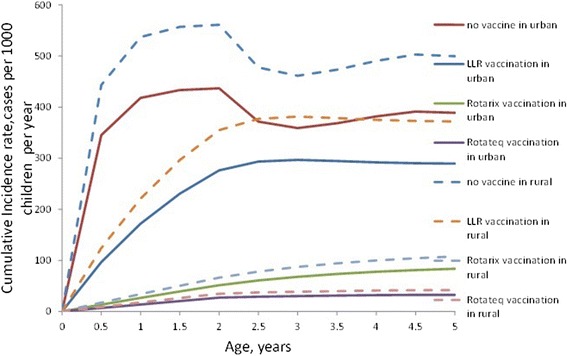
Impact of vaccination on the reduction of incidence by age

Figure 3 showed the simulated annual incidence of rotavirus infection by different vaccination options. Rotateq and Rotarix had significantly decreased incidence compared to LLR, particularly at children’s age between 0.5 to 2 years and particularly in the rural area . Both of the decreasing rates of the incidence caused by Rotateq and Rotarix in rural areas are 28.5 % higher than that in urban area [32]. The cumulative infection rate up to 5 years of Rotateq, Rotarix and LLR is 6.32, 12.05 and 64.26 %, respectively.

### Sensitivity analysis

**Fig. 4:**
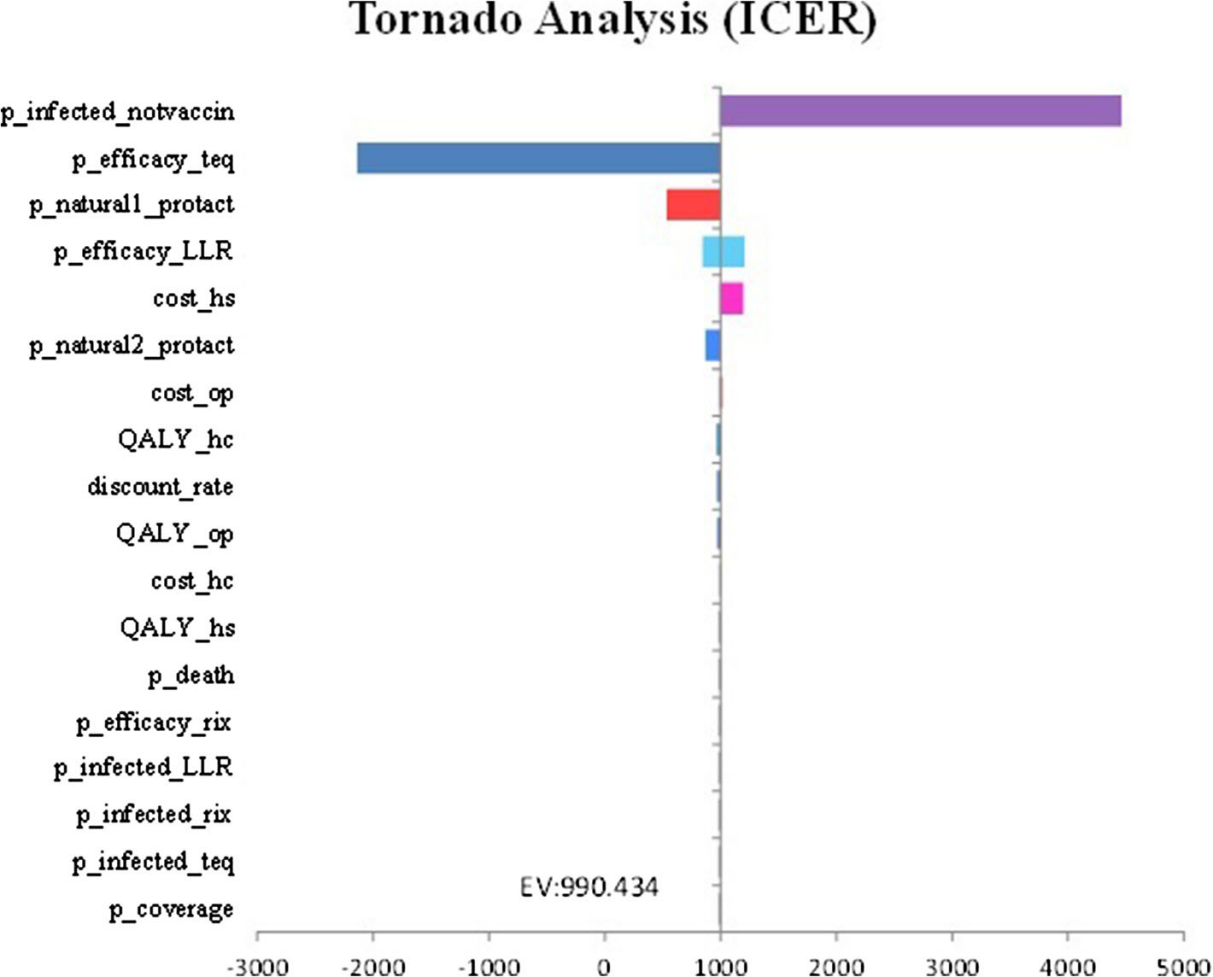
One-way sensitivity analysis: tornado diagram

**Fig. 5:**
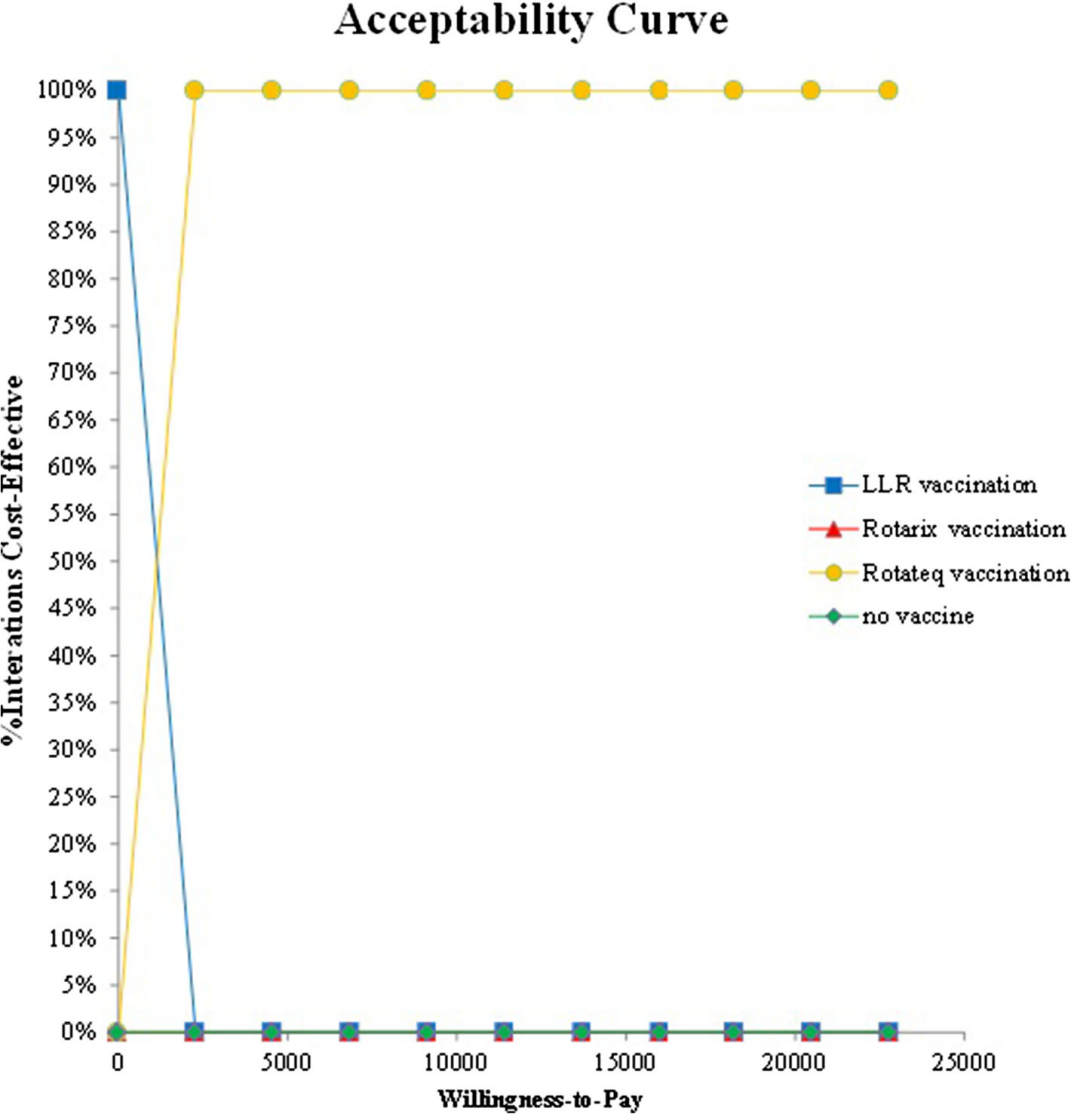
Acceptability curve

A tornado diagram showed results of one-way sensitivity analysis (Fig. 4). The ICER of Rotateq versus LLR was most sensitive to the incidence of rotavirus infection and secondly the vaccine efficacy. As a result of multivariate sensitivity analysis, the cost-effectiveness acceptability curve indicated that the probability of Rotateq to be cost-effective is nearly 100 % at WTP thresholds of $7594 (GDP per capita in 2015) (Fig. 5).

## Discussion

To best of our knowledge, this is the first economic evaluation to comprehensively compare all available options for rotavirus vaccination in China. In the hypothetical cohort of 100,000 infants, a national routine rotavirus vaccination by both Rotateq and Rotatrix showed high cost-effectiveness, and Rotateq reduced rotavirus disease burden most significantly, particularly among children aged between 6 months to 2 years and those living in the rural area. Considering the cost-effectiveness and the huge disease burden of rotavirus infection among children under 5 years, it is necessary to add vaccination against rotavirus into the current national routine immunization program.

Although LLR is the only approved vaccine in China and only Chinese-produced vaccines are regarded as a reliable supply for the national immunization program, the major problem of LLR is its complicated schedule, making it difficult to follow: one oral dose is given to the infant and children aged 2 to 35 months, followed by recommended yearly strengthening doses. Moreover, our study showed that compared to the two international vaccines, the effort of LLR to prevent and reduce rotavirus infection among children under 5-year is limited, particularly at children’s age between 0.5 to 2 years, at which 94 % of all episodes of rotavirus diarrhea occurred [4]. On the other hand, with the increase of age, the body's resistance gradually strengthens and the rate of rotavirus infection decreases [33]. Our findings is consistent with the report showing no significant protection among children vaccinated at 12–23 months of age [34]. For LLR, although earlier immunization and the administration of the full immunization regimen during infancy was recommended based on findings of a case-control study [28], the vague schedule makes confusion for parents and lead to difficulties in evaluation of the efficacy. Therefore, the current program is expected to scale up: based on the ICER shifting from LLR and health impacts on reduced disease burden of the two international vaccines, RotaTeq is dominating compared with Rotarix and expected to replace LLR. Besides the established safety and efficacy of the vaccine, the globally common rotavirus strains are the major cause of severe childhood diarrhea in China, suggesting that introduction of Rotateq vaccine would substantially reduce the disease burden [4]. In sensitivity analysis, infection rate affects the cost-effectiveness most significantly. After the introduction of rotavirus vaccines, rotavirus infection and hospitalizations among children has been reduced and in addition to such the direct effects, herd immunity also brings benefits to unvaccinated children [35]. However in China, due to relevantly low coverage, the impact of the vaccination on reduction of infection rate may be definitely limited, demonstrating that at the moment the national routine immunization against rotavirus with universal coverage would achieve good cost-effectiveness.

Without the national routine immunization program and public investment to reduce out-of-pocket payment to vaccination, it is extremely difficult to achieve universal coverage. The coverage of rotavirus vaccination is relevantly low, mainly attributable to the self-pay policy for the second-category vaccines, lack of knowledge of vaccination and rotavirus diseases among parents, and complicated and unclear schedule [36]. In China, vaccines are listed into two categories: the first category, freely and compulsorily provided, including bacillus Calmette-Guerin (BCG), hepatitis B vaccine, oral polio vaccine, measles vaccine and Diphtheria-Pertussis-Tetanus vaccine (DPT); and the second-category, which are totally self-paid. Under the current out-of-pocket payment policy for the second-category vaccines, the price for LLR is $24 per dose, and that for imported Rotateq or Rotatrix is uncertain and should be even higher, considering imported 7-valent pneumococcal vaccine costs as high as $140 per dose. Unlike most countries in the world, where financing for the immunization derived from either public funds or international donors and the user-fee is charged only a little, some vaccines addressing critical childhood diseases such as rotavirus infection and pneumonia are still self-paid and expensive, making profits for providers. As the results, the coverage of those vaccines is extremely low [36–38], not comparable to that of DPT as well as other first-category vaccines, which is generally regarded as a principle indicator for universal coverage of the vaccination, more than 90 % of the target children in China [39].

Our study has several limitations. First, regarding efficacy, data were derived from other settings and not specific for Chinese population for the two international vaccines, because both have not been approved in China yet. As for LLR, a unique rotavirus vaccine only approved and applied in China so far, due to no randomized controlled trial (RCT), data were derived from meta-analysis of domestic observational studies. Second, we did not include herd immunity effect in the model due to low coverage of the vaccination, potentially underestimated the effectiveness when adding the rotavirus vaccine into the routine immunization program. Third, as a model for decision making at the national level, we did not specifically consider the epidemiological characteristics at different geographical regions within the country, as the incidence of rotavirus infection in low-latitude provinces tended to be higher and likely to be affected by living habits, living environments, education level and vaccine coverage [40], raising as a further issue after scale-up of the current program.

## Conclusion

In China, rotavirus vaccination is highly cost-effective. To reduce disease burden of rotavirus infection, Rotateq is expected to replace LLR for the scale-up of the national routine immunization program, by increasing public investments and reducing costs of vaccines afforded by parents.

## Abbreviations

ACER: Average cost-effectiveness ratio
AGE: Acute gastroenteritis
BCG: Bacillus Calmette-Guerin
CPI: Consumer price index
DPT: Diphtheria-Pertussis-Tetanus
GDP: Gross Domestic Product
ICER: Incremental cost-effectiveness ratio
LLR: The Lanzhou Lamb rotavirus vaccine
ORS: Oral rehydration salts
PPP: Purchasing Power Parity
QALY: Quality adjusted life year
RCT: Randomized controlled trial
RV: Rotavirus
RVGE: Rotavirus gastroenteritis
WHO: World Health Organization
WTP: Willingness-to-pay
