# Correction to: Cost-effectiveness analysis of rotavirus vaccination in China: Projected possibility of scale-up from the current domestic option

**DOI:** 10.1186/s12879-018-3370-8

**Published:** 2018-09-27

**Authors:** Shuhui Cui, Ruoyan Gai Tobe, Xiuting Mo, Xiaoyan Liu, Lingzhong Xu, Shixue Li

**Affiliations:** 1 School of Public Health, Shandong University, Jinan, China; 2 Department of Health Policy, National Center for Child Health and Development, Okura 2-10-1, Setagaya-ku, Tokyo, 157-8535 Japan

## Correction

After the publication of our article [1] we have been made aware of a number of mislabelling and reporting errors, which were introduced in the preparation of the manuscript. The conclusions are not affected by these errors and thus remain unchanged.

### The corrections required are as follows

#### Correction 1

In the Methods section under the heading “Vaccine effectiveness”, the sentence:

“The protection effectiveness of Rotarix and Rotateq were derived from randomized controlled trials in other Asian regions such as Hong Kong, Taiwan and Singapore, considering the ethnic homogeneity [25], because there was no eligible data specifically for the Chinese population.”

has been corrected to:

“The protection effectiveness of Rotarix and Rotateq were derived from randomized controlled trials [25] and clinical reviews cited by economic evaluation studies in other Asian regions such as Hong Kong, Taiwan and Singapore, due to no eligible data specifically for the Chinese population.”

#### Correction 2

In the Results section under the heading “Health impacts and cost-effectiveness of vaccination”, the sentence:

“The total cost is even less than non-vaccination.”

has been corrected to:

“The ACER is even less than non-vaccination.”

#### Correction 3

Four corrections are required to Table 1 as follows:

Rotateq efficacy: the plausible range for sensitivity analysis “0 - 0.98” has been corrected to “0.883 - 1”; Source “38” has been corrected to “38, 42”.

“Mortality rate” under the heading “Parameters” has been removed as it appeared twice in the Table.

Costs for international vaccinations: the plausible range for sensitivity analysis “5 - 250” has been corrected to “50 - 250”

Infection rate: Source: “34” has been corrected to “21, 45”.

A corrected version of Table 1 appears below.

**Table 1:** ᅟ

	Baseline	Plausible range for sensitivity analysis		Sources
Parameters				
Discount rate	0.03	0	0.03	[31]
Vaccine coverage	25.3%	10%	28.6%	[36, 37]
Mortality rate	0.0058%	0.000029	0.000039	[41]
Rotateq efficacy	98%	0.883	1	[38, 42]
Rotateq infected	0.018%	0	0.00018	[42]
hospitalization1^a^	44%	0	0.44	[22]
Outpatient1 ^a^	28%	0	0.28	[22]
Home-care1 ^a^	28%	0	0.28	[22]
Rotarix infected	0.1%	0	0.001	[26]
LLR infected	0.9%	0	0.009	[41]
hospitalization3^c^	0.2%	0	0.002	[2]
Outpatient3 ^c^	7.9%	0	0.079	[2]
home-care3 ^c^	91.9%	0	0.919	[2]
Rotarix efficacy	96.1%	0.871	1	[25, 26]
LLR efficacy	72%	0.63	0.79	[27]
Infection rate	78.85%	0	0.7885	[21, 45]
home-care2b	32%	0	0.32	[22]
hospitalization2 b	33%	0	0.33	[22]
Outpatient2 b	35%	0	0.35	[22]
natural protact1d	77%	0	0.77	[23]
natural protact2 d	83%	0	0.83	[23]
Costs				
International vaccinations	200.00	50	250	[16, 17]
LLR vaccination	24			The national tariff
Hospitalizations	570.04	0	570.04	[43]
Outpatient	104.19	0	104.19	[43]
Home-care	11.52	0	11.52	[44]
Health Effects				
QALY(Hospitalization)	0.077	0.075	0.078	[30]
QALY(Outpatient)	0.081	0	0.081	[30]
QALY(Home-care)	0.082	0	0.082	[30]

#### Correction 4

The values presented in Table 2 were mislabelled and incorrectly shown. The correct version of Table 2 is shown below.

**Table 2:** Costs, health impacts and cost-effectiveness of rotavirus vaccines with comparison to no intervention

Strategy Name	Cost	QALYs	Incremental cost-effectiveness ratio ($/QALY)
No vaccine	2379.945	17.71296	(−)
LLR vaccine	2507.851	22.65899	0
Rotarix vaccination	5982.187	24.31454	2105.66
Rotateq vaccination	5577.902	24.44506	1715.14

#### Correction 5

The axes in Figure 2 were mislabelled. The correct version of Figure 2 is shown below.

**Fig. 2:**
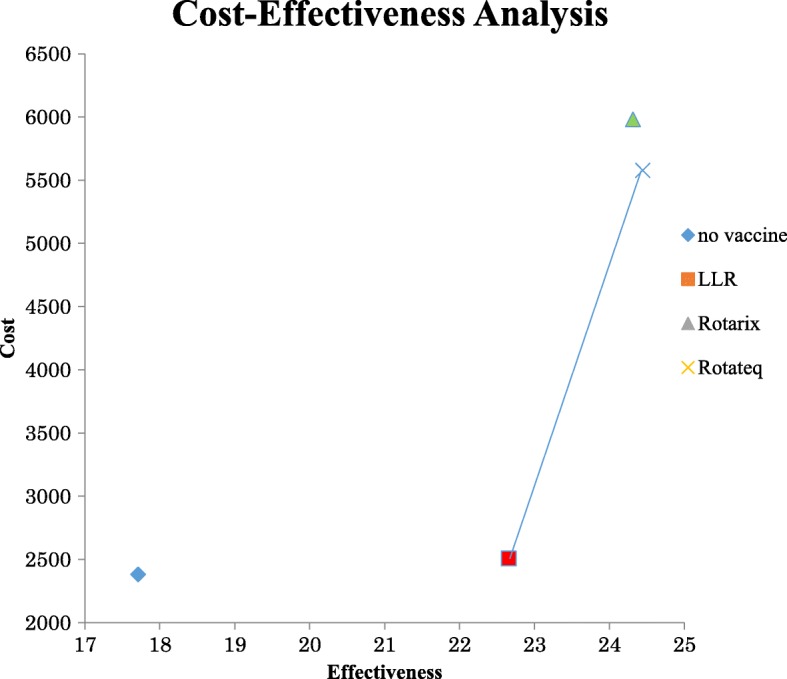
Cost-effectiveness of rotavirus vaccines at the baseline

#### Correction 6

The following reference should be included in the reference list:

45. Wu J, Yao Y, Hao W. Clinical Epidemiological Study on 244 Cases of Neonatal Rotavirus Infection. Chin J Nosocomiol, 1999, 9(4): 228–29 (in Chinese).
